# Perception and Initial Adoption of Mobile Health Services of Older Adults in London: Mixed Methods Investigation

**DOI:** 10.2196/30420

**Published:** 2021-11-19

**Authors:** Jing Pan, Hua Dong, Nick Bryan-Kinns

**Affiliations:** 1 College of Art and Design Nanjing Tech University Nanjing China; 2 Brunel Design School Brunel University London London United Kingdom; 3 School of Electronic Engineering and Computer Science Queen Mary University of London London United Kingdom

**Keywords:** older adults, mHealth, initial adoption, technology acceptance, design, mobile phone

## Abstract

**Background:**

Advances in mobile technology and public needs have resulted in the emergence of mobile health (mHealth) services. Despite the potential benefits of mHealth apps, older adults face challenges and barriers in adopting them.

**Objective:**

The aims of this study are to understand older adults’ perception of mHealth services and to discover the barriers that older adults face in the initial adoption of mHealth apps.

**Methods:**

This paper systematically analyzed main determinants related to mHealth services and investigated them through questionnaires, interviews, and a workshop. Two studies were carried out in London. In study 1, the questionnaires with follow-up interviews were conducted based on the literature review to uncover older adults’ perception (including perceived usefulness, perceived ease of use, and perceived behavioral control) of mHealth services. Study 2 was a workshop helping older adults to trial selected mHealth apps. The workshop was conducted by the first author (JP) with assistance from 5 research students. The barriers that older adults faced in the initial adoption period were observed. The interviews and workshop were audiotaped and transcribed. Descriptive statistics and the thematic analysis technique were used for data analysis.

**Results:**

In total, 30 older adults in London completed the questionnaires and interviews in study 1. The results of study 1 show that the lack of obvious advantage, low reliability, scary information, and the risk of privacy leakage would decrease older adults’ *perceived usefulness* of mHealth services; the design of app interface would directly affect the *perceived ease of use*; and aging factors, especially the *generation gap*, would create barriers for older users. In total, 12 participants took part in the workshop of study 2, including 8 who took part in study 1. The results of study 2 identified that *access to technology*, *the way of interaction*, *the risk of money loss*, *heavy workload* of using an mHealth app, and *different lifestyle* are influential factors to older adults’ adoption of mHealth services.

**Conclusions:**

The perceptions of mHealth services of older adults were investigated; the barriers that older adults may face in the initial adoption stage were identified. On the basis of the synthesis of these results, design suggestions were proposed, including *technical improvement*, *free trial*, *information clarification*, and *participatory design*. They will help inform the design of mHealth services to benefit older adults.

## Introduction

### Background

Owing to the development of information and communication technology (ICT), health care service delivery nowadays goes beyond traditional face-to-face interaction. ICT supports health care with electronic communication and system networking capabilities to provide, exchange, and facilitate exchange of health-related information [1]. Mobile health (mHealth) emerged in 2003; Robert Istepanian coined the term to describe the use of *emerging mobile communications and network technologies for health care* [2]. Compared with web-based health services delivered from desktops and laptops, mHealth services have the advantage of interacting with individuals with greater frequency and flexibility, without being limited by time and place [3]. Mobile technologies, especially smartphone-based apps, can improve the efficiency of health care delivery, ultimately make health care more effective [4-6] and help people to better control their chronic conditions [7,8]. However, despite the numerous benefits of mobile health (mHealth) apps, relatively little is known about whether older adults perceive that these apps confer such benefits. Their perspectives toward the use of mobile apps for health-related purposes have not yet been fully investigated [9].

“Living a healthier independent life” is vital for older adults’ quality of life [10]. Given that the aging population has become a global issue, making mHealth services more acceptable by older adults is of paramount importance. For instance, the World Health Organization has identified a good practice case study in Singapore’s Action Plan for Successful Aging, where a mobile app, *Healthy 365*, was successfully used [11].

### Prior Work

Although there has been a steady increase in the number of studies exploring technology adoption or acceptance among older adults, few have focused on mobile technologies, and even fewer have explored the acceptability of mobile technology use for health-related purposes [12]. Studies on mHealth adoption among older people are far less than those on general technology adoption among older people [13-15]. Because of the importance of, and the increased interest in the field, a scoping review protocol was proposed in 2020 to investigate the willingness, perceived barriers, and motivators in adopting mobile apps for health-related interventions among older adults [9].

Published studies on mHealth adoption [16-20] are mostly based on the technology acceptance model (TAM) [21] and its extended variations (ie, TAM2, TAM3, United Theory of Acceptance and Usage of Technology [UTAUT], and UTAUT2). The Health Belief Model (HBM) and the Protection Motivation Theory (PMT) also prove helpful in understanding mHealth adoption. In research on health behavior, eHealth literacy [22], self-efficacy [23], perceived vulnerability, perceived severity, and health consciousness [24] are listed as influential factors in people’s adoption of health information technologies. Sun et al [25] integrated several models to find that users’ intention to use mHealth services was determined by 5 key factors: performance expectancy, effort expectancy, social influence, facilitating conditions, and threat appraisals.

Deng et al [18] extended the TAM with *trust* and *perceived risks* in studying mHealth adoption in China. Alam et al [19] extended UTAUT to include *perceived reliability* and *price value* to investigate mHealth adoption in Bangladesh. These studies used quantitative methods (eg, survey questionnaires) and recruited patients from local hospitals. Cajita et al [26] investigated the intention to use mHealth in older adults with heart failure, and associated facilitators and barriers [12], using mixed-methods (ie, large survey + small-scale interview). Minimal qualitative research was conducted with *well-old users* [27] who are the largest potential beneficiaries of mHealth services.

Previous research has mainly investigated how older adults use technologies before the objectification phase and usability problems after the conversion phase [28], and few have investigated the initial adoption stage, that is, using only elementary features and limited functions of mobile technologies. Grindrod et al [29] evaluated user perceptions of 4 mobile medication management apps with older adults (those aged ≥50 years) through usability testing and found that most participants *were frustrated by their initial experiences with the apps*.”

This paper fills these gaps by exploring older adults’ initial adoption of mHealth apps, using qualitative questionnaires combined with interviews and user trial workshops to reveal their perceptions and contextualized experiences. The insights help generate design suggestions to make mHealth services more acceptable to older adults.

### Theoretical Framework

mHealth services use ICT. They are relevant to technology adoption theories and can be traced back to the theory of reasoned action (TRA) [30]. On the basis of the TRA, Davis et al [31] developed the TAM in which they suggested that *perceived usefulness* and *perceived ease of use* are the 2 most important individual beliefs about using information technology. Other researchers have extended the TAM and proposed the TAM2 [21] and the TAM3 [32], decomposing *perceived usefulness* and *perceived ease of use*. Ajzen [33] developed the Theory of Planned Behavior (TPB) to extend the TRA and added the new construct of *perceived behavioral control*. Venkatesh et al proposed the UTAUT [34], combining 8 existing theories, and the UTAUT2 [35] emphasized the consumer use context.

As a kind of health behavior, mHealth adoption is also relevant to theories of health behavior, such as the HBM [36] and PMT [37]. The HBM hypothesizes that health-related behavior depends on the combination of perceived susceptibility, perceived severity, perceived benefits, perceived barriers, cues to action, and self-efficacy. The PMT stems from both threat appraisal (perceived vulnerability and perceived severity) and coping appraisal (response efficacy, self-efficacy, and response cost) processes.

The theoretical framework of this study is based on the TRA, TAM, TPB, UTAUT2, HBM, and PMT (column 2 in Table 1). Eight main constructs (column 1 in Table 1) were extracted by grouping similar factors in these models. These constructs will be further investigated through primary studies in order to gain insights into older adults’ perceptions and the initial adoption of mHealth services.

**Table 1:** Eight main constructs extracted from existing models.

Construct	Definition	Origin from existing models
PU^a^	An individual’s perception that using a particular system would enhance his or her job performance [38].	Perceived usefulness in TAM^b^ Performance expectation in UTAUT2^c^ Perceived benefits in HBM^d^ Response efficacy in PMT^e^
PEOU^f^	An individual’s perception that using a particular system would be free of effort [38].	Perceived ease of use in TAM Effort expectancy in UTAUT2 Perceived barriers in HBM
PBC^g^	An individual’s perception of how easy or difficult it will be to perform the target behavior [33]. The perceptions of internal and external constraints on behavior and encompasses self-efficacy, resource facilitating conditions, and technology facilitating conditions [35].	Perceived behavioral control in TAM Facilitating conditions in UTAUT2 Perceived barriers and self-efficacy in HBM Self-efficacy and perceived cost in PMT
SI^h^	An individual’s perception of the degree to which most people who are important to him or her approve or disapprove of the target behavior [30].	Subjective norm in TRA^i^ Subjective norm in TPB^j^ Social influence in UTAUT2
HM^k^	An individual’s perception of the fun or pleasure derived from using a technology [35].	Hedonic motivation in UTAUT2
PV^l^	An individual’s cognitive tradeoff between the perceived benefits of the applications and the monetary cost for using them [39].	Price value in UTAUT2
HB^m^	The extent to which an individual tends to perform behaviors automatically because of learning [40]. Habit is a perceptual construct that reflects the results of prior experiences [35].	Habit in UTAUT2 Experience in UTAUT2
PHC^n^	An individual’s perception of the risk of acquiring an illness or disease [37] and the seriousness of contracting an illness or disease [36]	Perceived susceptibility and perceived severity in HBM Perceived vulnerability and perceived severity in PMT

^a^PU: perceived usefulness.

^b^TAM: technology acceptance model.

^c^UTAUT2: United Theory of Acceptance and Usage of Technology.

^d^HBM: Health Belief Model.

^e^PMT: Protection Motivation Theory.

^f^PEOU: perceived ease of use.

^g^PBC: perceived behavioral control.

^h^SI: social influence.

^i^TRA: theory of reasoned action.

^j^TPB: Theory of Planned Behavior.

^k^HM: hedonic motivation.

^l^PV: price value.

^m^HB: habit and experience.

^n^PHC: perceived health condition.

## Methods

### Overview

**Figure 1:**
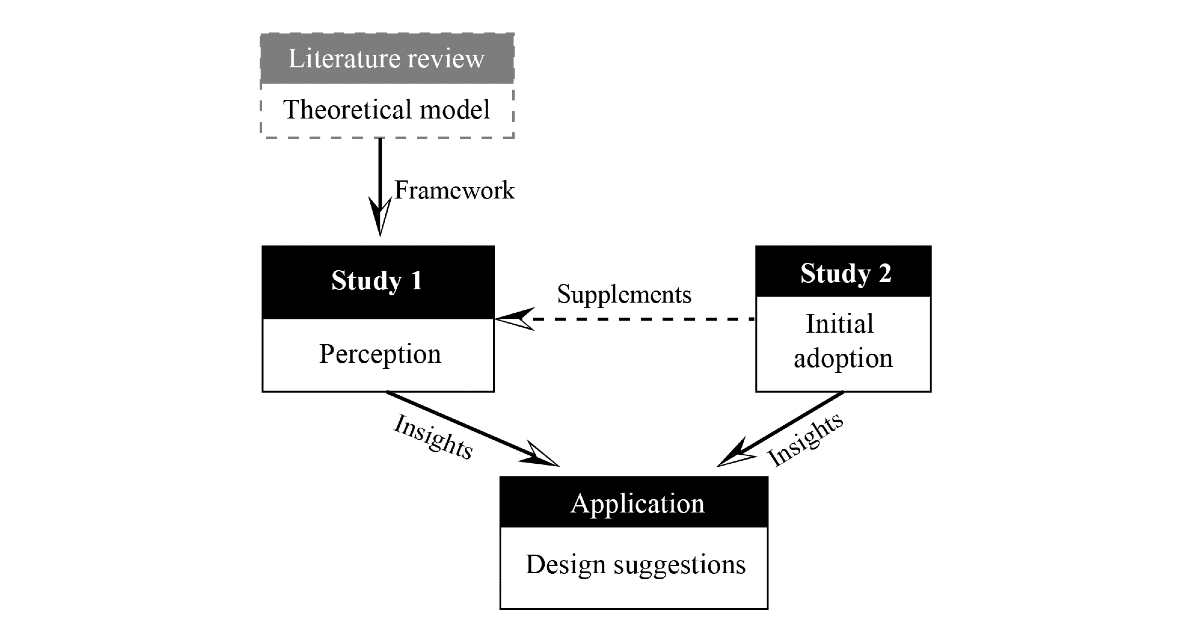
An overview of this research.

An overview of this study is shown in Figure 1. Study 1 investigated older adults’ perception of mHealth devices through questionnaires and interviews based on a literature review. Study 2 observed how older adults initially use mHealth apps to identify the barriers and experiences they have in mHealth adoption.

The research received ethical approval from the Queen Mary University of London (QMERC2016/31). The insights from these 2 studies help generate design suggestions to make mHealth services more acceptable to older adults.

### Study 1: Investigation of Perceptions

Study 1 was conducted between January and February 2017 in London. The study comprised a 15-minute questionnaire and a follow-up interview (approximately 30-45 minutes). Conducting face-to-face interviews following questionnaires can not only help to obtain more detailed information from the participants but also help rectify any misunderstanding of the answers. The mHealth service discussed here mainly focused on health-related services that can be accessed by smartphones and tablets, for example, websites and mobile apps.

Existing studies have proved that age plays a moderating role in mHealth adoption [41-43] and factors have different impacts on mHealth adoption intention among different age groups [23]. In Britain, old age can be *any age after 50* years and this definition has been adopted in many human computer interaction studies and initiatives such as age-friendly cities. In this study, we recruited *well-old users* [27] aged between 50 and 70 years in East London. People with serious disease or impairments and aged ≥70 years were excluded; this was to ensure independent participation in the study (requiring traveling and basic understanding of digital technology).

We targeted 30 samples, as suggested by Corder and Foreman [44]. We included all 32 older adults who contacted us, but 2 of them failed to complete the whole process, so the valid responses were 30. Convenience sampling was used; it is cost-effective and has been widely accepted in information system research [45]. Participants were recruited from the Age UK, Hackney Mobile Centre, and the Queen Mary University of London. The questionnaires and interviews were completed in the classrooms of Age UK East London, Hackney Mobile Centre, or the Senior Common Room of Queen Mary University of London, depending on the time and venue availability. The details of study 1 are shown in Table 2.

**Table 2:** Details of study 1.

Construct	Content	Research item
Demographic information	Age, gender, living arrangement, education level, and employment status	Questions 1-5
PHC^a^	Perceived health condition	Question 6
PBC^b^	Facilitating conditions (access to technology) Age-related changes in using mobile technology	Questions 7-8 Questions 12-17
HE^c^	Using different devices for health purposes Using mobile devices for different purposes	Question 10 Question 11
PU^d^	PU of web-based health information PU of mobile devices on health and well-being PU of mobile health apps	Question 9 with interview Question 18 with interview Questions 19-31 with interview
PEOU^e^	Perceived ease of use	Interview

^a^PHC: perceived health condition.

^b^PBC: perceived behavioral control.

^c^HE: habits and experience.

^d^PU: perceived usefulness.

^e^PEOU: perceived ease of use.

To understand older adults’ perceived usefulness of mHealth apps, the participants were asked to rate the usefulness of different features of mHealth apps and to give reasons for low scores. An mHealth app typically offers more than one function; in other words, an mHealth app has multiple features. To understand main features offered by typical mHealth apps, the authors (JP and HD) searched for the term *health* in both the App Store (iOS system) and Google Play Store (Android system) in December 2016, downloaded the top 50 health-related apps in each system, and analyzed the features of each app. For example, Apple Health has features for *fitness and exercises* and *for emergency (providing vital medical information of you in an emergency)*. As a result, 13 features were extracted from the existing health-related apps, and they were evaluated by the older adults participating in study 1.

To understand older adults’ *perceived behavioral control* of using mHealth services (eg, mobile apps), the participants were asked to rate how different age-related changes might stop them from using an app, for example, *visual impairment*, *hearing loss*, *decline in memory*, *decline in the ability to understand written and spoken languages*, *decline in the ability to focus attention*, and *decline in movement control* [46]. *Generation gap* was also added, as we found from our previous pilot study that older adults had difficulties in understanding new terms generated by the younger generation. For example, they were confused by the *menu* or *navigation* of a digital interface.

### Study 2: Observation of Initial mHealth Adoption

This study took place as a workshop in March 2017 at the Hackney Mobile Centre in East London, where a Wi-Fi connection was available. mHealth apps were introduced to older adults, and they were helped to start using these apps. At the same time, how they initially used mHealth apps was observed to identify the barriers and experiences that older adults have in mHealth adoption. mHealth apps were selected from the App Store and Google Play Store. After reviewing over 100 mHealth apps, we identified 4 categories beneficial to older adults’ health, namely *web-based diagnosis*, *step tracker*, *calories calculator or food diary,* and *health monitor*.

As Google Fit (Android system only) and Apple Health (iOS only) are embedded in most smartphones, they were also included in the trial. An additional 4 pairs of apps, free and available in both Android and iOS systems, were chosen for each category (Table 3).

**Table 3:** Ten apps introduced in the workshop (in 5 pairs).

Pair 1	Pair 2	Pair 3	Pair 4	Pair 5
Embedded health platforms	Diagnosis on the web	Step tracker	Calories calculator and food diary	Health monitor
Google Fit 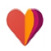	Health Tap 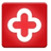	Movesum 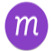	Lifesum 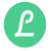	iCare Health Monitor 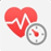
Apple Health 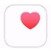	Babylon Health 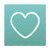	Pacer Health 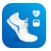	My Fitness Pal 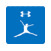	mySugr Diabetes Diary 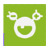

#### Pair 1: Embedded Health Platforms

Google Fit (Android system) and Apple Health (iOS system) apps are often embedded in users’ smartphones. They have the basic function of step counting and integrating health information from third-party health apps in the users’ phones or wearable devices to track fitness, nutrition, sleep, and weight.

#### Pair 2: Web-Based Diagnosis

The Health Tap and Babylon Health apps enable users to have online consultations with physicians and health care professionals via SMS text messaging and video messaging. They also help make appointments with general practitioners (GPs) or pharmacies in certain locations. Primary consultancy is free, while more professional and responsive services incur extra costs.

#### Pair 3: Step Tracker—Movesum and Pacer

The main function is to automatically record the user’s steps, distance, active time, and calories burned all day. Movesum motivates people to do exercise by showing what food they have *burned* while Pacer allows people to join different online groups based on common health goals and interests. Both apps use smart notifications to help users reach their daily goals.

#### Pair 4: Calories Calculator and Food Diary

Unlike the s*tep trackers*, the Lifesum and My Fitness Pal apps import activity information from other apps and focus more on what people eat. Both provide barcode scanners for easy food tracking, recording, and evaluating people’s diets. They also give diet or exercise suggestions, but, to obtain personalized suggestions, users need to upgrade to a premium version that requires extra payment.

#### Pair 5: Health Monitor

The iCare Health Monitor app measures blood pressure, heart rate, vision, hearing, and SpO2breath rate without extra devices. The mySugr Diabetes Diary app includes a blood sugar tracker, carb logger, and a bolus calculator (Europe only). After users put in their meal and medical information, together with activity information from other apps, it will show the estimated glycated hemoglobin level (an objective measure of glycemic control). Users can export their daily, weekly, or monthly medical analysis and report them with a paid version.

The workshop was conducted as an event at the Hackney Mobile Centre. Participants were recruited through the Hackney Mobile Centre’s group email contact and poster advertisement. The recruiting criteria were age between 50 and 70 years, using a smartphone, and being interested in mHealth apps. In total, 21 older adults contacted us for participation; however, considering the size of the venue and the number of researchers, we recruited only 57% (12/21). Older adults who took part in study 1 were prioritized; 8 older adults from study 1 participated in the workshop, and 4 more participants were selected according to the order in which they contacted us. All participants were asked to bring their own smartphones. The workshop lasted 2 hours. All the 10 free mHealth apps were introduced to all participants. They were then invited to decide on which app to be downloaded to their own phones based on their interests.

The first author (JP) organized and conducted the workshop with the assistance of 5 research students. The research students were recruited as volunteers through the university’s group email contact with the following criteria: (1) have experience in communicating with older adults, (2) native English speakers, (3) interested in mHealth apps, and (4) have a smartphone that can install at least five of the selected apps. The research students were asked to download and try each selected app the day before the workshop. They were trained by JP 1 hour before the start of the workshop, and all followed the same procedure: each was equipped with a record sheet template to tick the apps tried and to record demographic information, negative and positive perception, reasons for giving up, and willingness to use the app in the next 3 months. Each of the research students and JP took care of 2 older participants, sitting in between them, helping download apps, taking notes, and making audio recordings. After the workshop, JP collected all the notes and audio recordings and discussed with each research student about their observation of the workshop. JP transcribed the notes immediately after the workshop and checked the accuracy of the notes with each research student through email communication.

Descriptive statistics were used to summarize the participants’ characteristics and outline the general situation of mHealth adoption among older adults in London. Qualitative data from interviews and workshops were analyzed using the thematic analysis method. The 6-step thematic analysis approach by Braun and Clarke [47] was adopted. A hybrid process of inductive and deductive coding [48] was applied to continually reflect on and refine the themes. Quotes from participants were referenced to support the research statements.

## Results

This section reports the outcomes from study 1 and study 2.

### Outcomes of Study 1

The 30 participants completed both the questionnaire and the follow-up interview. The sample characteristics of study 1 are shown in Table 4. The participants were asked to rate their own perceived health condition from 1 to 5 points (1 for *poor* and 5 for *excellent*). The average score of all the participants was 3.7 points (SD 1.15 points; minimum=1 point, maximum=5 points); 66% (20/30) of them had a positive perception (scores 4-5) of their own health.

**Table 4:** The sample characteristics of study 1.

Characteristics		Values, n (%)^a^
**Age (years)**		
	50-54	12 (40)
	55-59	6 (20)
	60-64	5 (17)
	65-70	7 (23)
**Gender**		
	Male	17 (57)
	Female	13 (43)
**Living arrangement**		
	Alone	10 (33)
	With partner only	6 (20)
	With child only	3 (10)
	With partner and child	7 (23)
	With other relative	1 (3)
	Other	3 (10)
**Education level**		
	Postgraduate or higher degree	11 (37)
	First Degree	4 (13)
	HND^b^, HNC^c^, or teaching	2 (7)
	BTEC^d^ or college diploma	7 (23)
	Associate level	3 (10)
	Lower degree	3 (10)
**Employment status**		
	Retired	7 (23)
	Employed part-time	5 (17)
	Employed full-time	9 (30)
	Unemployed	9 (30)

^a^There were a total of 30 valid samples.

^b^HND: Higher National Diploma.

^c^HNC: Higher National Certificate.

^d^BTEC: Business and Technology Education Council.

#### Access to Technology

Among all the participants, all (30/30, 100%) had access to the internet, 80% (24/30) had a PC, 47% (14/30) had a cell phone (simple mobile phones), 80% (24/30) had a smartphone, 67% (20/30) had a personal tablet, and 7% (2/30) had smart wristbands. In total, 80% (24/30) of participants had a smart mobile device capable of searching on the web and installing apps.

#### Using Different Devices for Health Purposes

In total, 13% (4/30) of the participants used an app related to health. The apps used were Fitbit, GoogleFit, Runkeeper, and Apple Health. Their adoption of mHealth apps was rather passive, as they stated:

I use it because it [is] just there, the information turns out automatically, so I can see it.

My daughter bought the wristband for me, so I wear it. But rarely check the data on the phone.

**Figure 2:**
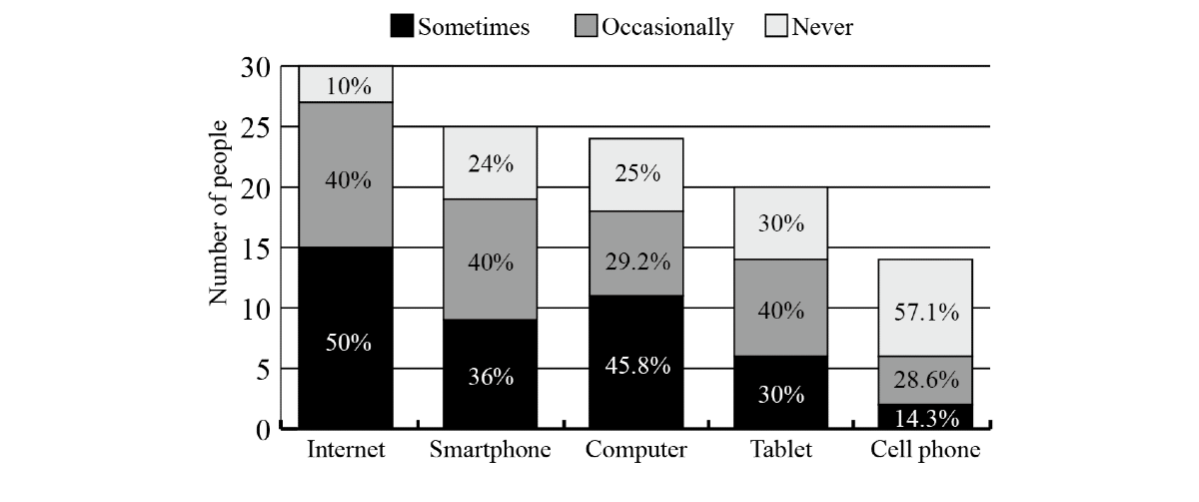
Frequency of using the internet and different devices for health purposes by older adults.

We also investigated how frequently the participants used the internet and different devices for health purposes. The results are shown in Figure 2.

All participants had access to the internet, and 90% (27/30) of them had experience in using the internet for health purposes. Not many people use mHealth apps; smartphones seem to be the first choice for older adults to obtain health information or services on the web. In total, 76% (18/24) of the participants had smartphones and accessed health information or services by their smartphones.

#### Using Mobile Devices for Different Purposes

Mobile devices are required to adopt mHealth services. Therefore, we investigated how older adults use mobile devices. The results are summarized in Table 5 (excluding people who only have a simple cell phone as their devices may have limited their choices).

**Table 5:** Frequency of using mobile devices for different purposes.^a^

Purpose of use	Frequency of use^b^		
	Values, Minimum	Values, Maximum	Values, mean (SD)
Creation (eg, taking a photo, filming a video, or editing a file)	2	5	3.5 (0.9)
Traffic and transportation (eg, Google Maps and Citymapper)	1	5	3.3 (1.3)
Social engagement (eg, Facebook or Twitter)	1	5	2.8 (1.6)
Entertainment (eg, playing games, listening to music, and watching videos)	1	5	2.7 (1.5)
Health and fitness (eg, searching information, sport tracking, and health management)	1	4	1.9 (1.1)
Web-based transaction (eg, web-based shopping, banking, and paying bills)	1	4	1.8 (1.2)

^a^The valid sample size is 24.

^b^1=never; 2=less than once a month; 3=every month; 4=every week; 5=every day.

#### Perceived Usefulness of mHealth Services

As few participants had experience using mHealth apps or wearable health devices, we asked how older adults perceived the usefulness of mobile devices for their health and well-being. The main health-related benefits are seeking information on health issues (70%), making appointments, and maintaining contact with physicians (67%). However, 75% (22/30) of the participants did not think that mobile devices were beneficial to their health or had doubts. As participants said:

I don’t know who put the health information online, maybe someone is just pretending to be a specialist.

Same symptoms on different people can be result from different reasons and same recipe may have different effect on different people, even physicians cannot give me suggestions before seeing me face to face.

If they had to search for health information on the web, most of the older adults would choose the website of the National Health Service, and some also said that they would search for academic articles to obtain more reliable information.

To understand older adults’ perceived usefulness of mHealth services, the participants were asked to evaluate 13 different functions using a scale from 0 to 4 (0 means *this function is not useful at all* and 4 means *this function is very useful*). The most highly valued function was *for emergency* (mean 2.83, SD 1.40) followed by *making an appointment with physicians or hospitals or GPs* (mean 2.79, SD 1.50) and *knowledge about health and health preservation* (mean 2.54, SD 1.39). Some respondents also mentioned that they would try to *communicate with a physician on the web* only if they were unable to go outside. Most of them thought that the mHealth service was not bad but not essential. As one participant noted:

It is a good service, but not necessary to me. I’m satisfied with life without it.

The main reasons for the lower scores (negative perceptions) are summarized in Table 6.

Four main factors that decrease the perceived usefulness of mHealth services were identified:

1. No obvious advantage: compared with older adults’ own way of taking care of themselves, the mHealth service did not seem to show sufficient advantages for them.
2. Low reliability: the information or result provided by the mHealth service did not have or show high reliability.
3. Scary information: health information can be difficult to understand or scary to know to some people.
4. Risk of privacy leakage: the concern about privacy has hindered older adults from putting their personal information on their mobile phones or on the internet.

**Table 6:** Negative perception of mobile health services.

Function	Reasons for giving a low score
Knowledge about health and health preservation information	“I don’t trust it.”
Self-assessment or self-diagnosis (eg, check health statues with apps or websites by yourself)	“I’m not a health professional, I prefer to see a physician.” “Pharmacy is just around the corner, why should I do it myself?” “I rarely do self-diagnose or assessment, the thinking if there’s something wrong with me will make people really sick.”
Health measurement (eg, body temperature, blood pressure, blood glucose, and heartbeat)	“I’m afraid that I can’t use it in a right way and that will make the measurement not accurate.” “I don’t want to buy all the devices for measurement.”
Access to health record or history	“I don’t really understand all the terms, there’s no need for me to see it.” “Looking into the bad record makes me feel even worse.”
Making an appointment with physicians or hospitals or GPs^a^	“Calling the GP is easy, using an app for it may make it more complicated.”
Helping with healthy diet (eg, healthy recipes, calories calculator, or food diary)	“It’s hard to calculate the calories or sugar in an accurate way.” “I don’t think I can keep on with the diary.” “I’m already eating in a quite healthy way.”
Information of medicine	“I can check it on the package.”
Fitness and exercises (step counter and exercise guide)	“I don’t need it.” “I’m not an exercise person.” “The number is not accurate.”
Communicating with a physician on the web	“I like seeing people’s eyes.” “I feel more comfortable to talk with a physician face to face.” “Physicians cannot see and feel how I am web-based.” “Although you have communication with a physician web-based, he or she will always suggest you to come to the GP.” “You will still have to go to the GP or hospital for some tests.”
Communicating with people who have the same health issue	“I don’t want to talk about my disease with strangers.” “Same symptoms on different people can be result from different reasons and same prescription may have different effect on different people. They are not specialist, there’s no meaning to discuss with other patients.”
Long-term situation management	“I don’t have serious long-term situation.” “My diabetes is under control and I don’t think I need an app to deal with it.” “I think going to see the physicians regularly is the best way to control my long-term situation.”
Reminder for taking medicine or meeting a physician	“I don’t take medicine.” “My GP will send me a message to remind me of the appointment.”
For emergency (eg, calling for help automatically or providing vital medical information of you in an emergency, such as allergies and medical conditions)	“I don’t want my information to be seen by others, what if I lost my phone?”

^a^GP: general practitioner.

#### Perceived Ease of Use of mHealth Apps

To understand what really affects older adults’ perceived ease of use of apps, the participants were asked the following questions:

What is “ease of use” of an app to you?

Which of these two apps you use is “easier to use” and why?

The factors identified were *clarity of the language*, *text size*, *knowing where (which icon or button) to press*, *knowing what the icon or button means*, *finding what I need easily*, *knowing how to use without learning*, and *having no problem to do what I want*.

#### Perceived Behavioral Control of Using a Mobile App

In the questionnaire, participants ranked how aging factors might stop using an app. The higher the score, the greater the influence. The results are presented in Table 7.

**Table 7:** How aging factors influence older adults’ adoption of mobile apps.^a^

Aging factors	Influence^b^		
	Values, Minimum	Values, Maximum	Values, mean (SD)
Generation gap (having difficulty to understand the new terms generated by the younger generation)	0	4	1.7 (1.3)
Visual impairment	0	4	1.5 (1.4)
Decline in memory	0	4	1.5 (1.3)
Decline in the ability to understand written and spoken languages	0	4	1.2 (1.5)
Decline in the ability to focus attention	0	4	1.1 (1.3)
Hearing loss	0	4	0.9 (1.0)
Decline in movement control (eg, typing or clicking)	0	4	0.9 (1.1)

^a^The valid sample size is 30.

^b^0=no influence; 1=small influence; 2=some influence; 3=big influence; 4=great influence.

*Generation gap* has the most influence on older adults’ adoption of mobile apps. Visual impairments have the second biggest influence, followed by Decline in memory.

### Outcomes of Study 2

The workshop (study 2) was conducted in March 2017, a month after the completion of study 1. In total, 12 participants (5/12, 42% males and 7/12, 58% females), aged between 50 and 70 years (minimum 52, maximum 66; mean 56.8, SD 4.5) participated in the workshop. Table 8 shows the sample characteristics of study 2.

**Table 8:** The sample characteristics of study 2.^a^

Characteristics		Values, n (%)
**Age (years)**		
	50-54	2 (16)
	55-59	5 (42)
	60-64	4 (33)
	65-70	1 (8)
**Gender**		
	Male	5 (42)
	Female	7 (58)
**Living arrangement**		
	Alone	2 (16)
	With partner only	6 (50)
	With child only	3 (25)
	With partner and child	1 (8)
	With other relative	0 (0)
	Other	0 (0)
**Education level**		
	Postgraduate or higher degree	0 (0)
	First degree	1 (8)
	HND^b^, HNC^c^, or teaching	2 (16)
	BTEC^d^ or college diploma	5 (42)
	A-level	4 (33)
	Lower degree	0 (0)
**Employment status**		
	Retired	7 (58)
	Employed part-time	3 (25)
	Employed full-time	0 (0)
	Unemployed	2 (16)

^a^The valid sample size is 12.

^b^HND: Higher National Diploma.

^c^HNC: Higher National Certificate.

^d^BTEC: Business and Technology Education Council.

#### Barriers to the Initial Adoption of mHealth Apps

Embedded health platforms proved the easiest for participants to try because of the lack of need for downloading; 1 participant abandoned the tests when downloading a new app; there was not enough storage space in her phone. She said:

It says there’s not enough space. I have to delete old apps to install new apps. But I am not sure if I really want this one [the app introduced in the workshop]

In total, 2 participants withdrew from the tests during installation. When the app asked for access to their location or photos, they gave up, worrying about the security of their personal data:

Why they want to access my camera? I don’t want to share my location. It’s unsafe. I’d rather not use it.

A total of 2 participants decided to quit the tests during the registration process. Almost all health-related apps require registration, which often requires personal information such as age, gender, and weight. Participants felt that their privacy was invaded, especially when they had no idea what these apps could do for them. One participant complained:

It asked for too much. You need to be cautious when putting personal information online. . .never know who is on the other side of the app. Of course, if it can really benefit my health, I’ll take that. But for now, I just want to have a try, I don’t know if it is what I want.

**Figure 3:**
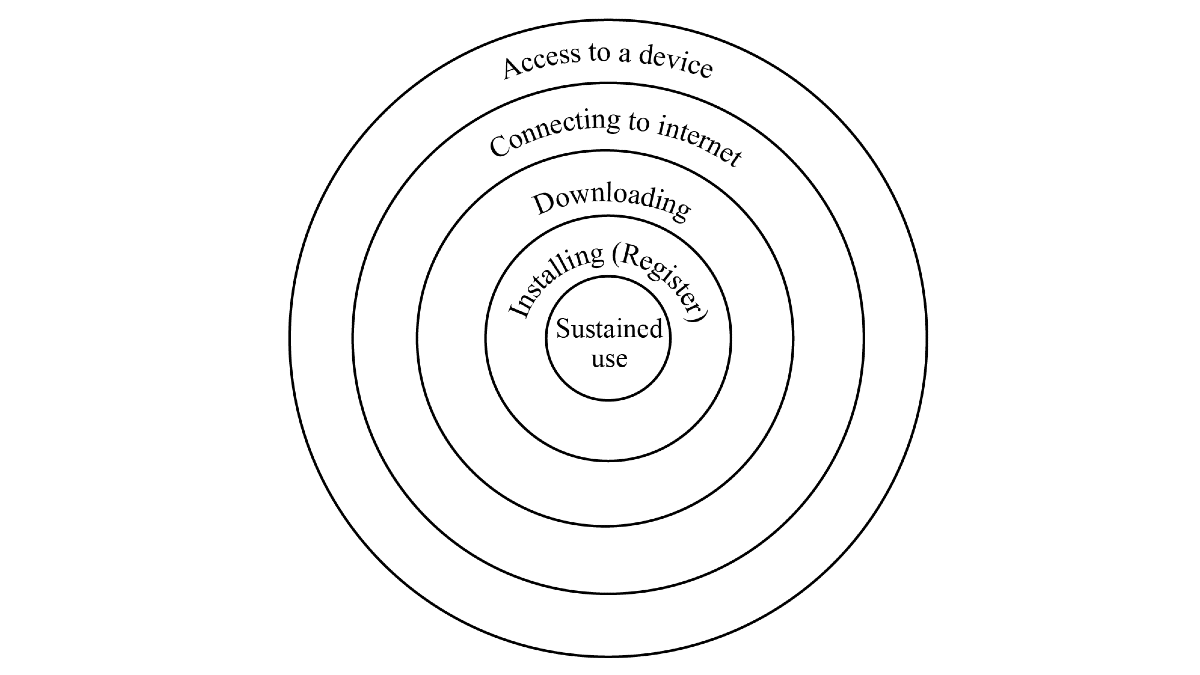
Barriers to adopting mobile health apps.

The physical barriers to mHealth adoption are illustrated in Figure 3 based on these observations. First, older adults must have access to a mobile device with adequate space for app installation. Second, the internet must be available (meaning that people are willing to pay for using mHealth apps and are comfortable with connecting their devices to the internet). Third, people will choose an mHealth app to download and then install the app. Registrations are often required after the installation of an mHealth app.

#### Feedback From Initial Experience

In total, 58% (7/12) of participants installed one or more mHealth apps in the workshop. The feedback on their initial experience is summarized in Table 9.

From the participants’ feedback, factors influencing older adults’ initial experience of using mHealth apps were identified as follows.

**Table 9:** Participants’ feedback regarding their initial experience of mobile health apps.^a^

App categories	People who tried, n (%)	Feedback
Embedded health platform	9 (75)	“I’m not really using it, I just notice my steps when the notification from the app shows up.”
Diagnose on the web	4 (33)	“It keeps asking me to put in personal information before I can find out if I really want this.” “If I… will it cost my money?” “It’s useless; it still asked me to see a physician.” “There’s no response.” “It requires very good internet connection”. “I won’t do a face chat without Wi-Fi.”
Step tracker	3 (25)	“It (Pacer) doesn’t have much difference with Google fit” “I’m not eating junk food, showing me how much junk food I have burnt is useless.”
Calories calculator and food diary	6 (50)	“It keeps asking me to put in personal information before I can find out if I really want this.” “I don’t have patience to calculate my calories every day.” “Scanning bar codes for recording calories is cool, but many self-made food still need to be calculated by myself.” “If the calculation is not accurate, it isn’t helpful to me.”
Health monitor	7 (58)	“The way to use it is amazing!” “I don’t want to buy any extra device unless it’s really accurate and not very expensive.”

^a^The valid sample size is 12.

#### Access to Technology

Access to technology can not only stop people from adopting an mHealth app, but also affect their experience in using it. For example, when web-based consultancy was introduced, 1 participant mentioned the following:

I don’t have Wi-Fi connection at home, and I won’t do a face chat without Wi-Fi. Otherwise, I’ll pay very expensive Internet fees. So actually, this function is not useful to me.

Some participants had an unnerving experience in the self-diagnosis process. Long waiting for system responses often frustrates people when the responses come slowly owing to the unstable connection of the internet or low speed:

It doesn’t respond.

My phone is stuck. What’s wrong with it?

What should I do now?...Should I keep waiting?

#### The Way of Interaction

A one-way interaction may fail to attract older adults’ attention and thus has little impact on their health. This was observed most obviously in the *embedded health platform* and *Step Tracker.* Before this workshop, the participants who used the platforms were unaware that they were using an mHealth app. As one participant noted:

I’m not really using it. I just notice my steps when the notification from the app shows up

Without connection with other health-related apps, the platforms mostly work as a *Step Tracker*. People who tried this type of app in the workshop did not show much enthusiasm. One participant said:

I can find that I walk more or less steps than yesterday by using this app. I see it [the numbers], but I do care about it.

*iCare Health Monitor*, an app used to measure blood pressure, heart rate, vision, hearing, SpO2, and breathing rate was the most welcomed app in the workshop; 7 (58%) participants tried this app and were surprised to be able to measure their blood pressure using a phone camera. Although they were told that the measurements might not be very accurate, all of them intended to use this app in the next 3 months.

#### Risk of Money Loss

The participants were not ready to pay for a mobile health service that they did not understand. Many participants kept asking questions such as:

Is it free?

If I...will it cost my money?

This was observed in *web-based diagnosis* apps. With these apps, users can perform a self-diagnosis step-by-step or consult a physician or therapist and receive medical advice quickly; 33% (4/12) of participants tried an app in this category. Although they had been told that *Talking to a qualified physician on demand via a video consultation or phone call* will cost money while *Texting your medical questions to a physician to receive a quick, personal response* is free, they were reluctant to use this free function, worrying about wasting money by misuse.

#### Heavy Workload

Excessive workload prevents older adults from using mHealth services. This was the case for the *calories calculator and food diary.* These apps require users to enter a large amount of information every day to obtain accurate results. One participant noted:

This will work only if I put accurate data into it. It’s difficult to count calories of what I eat. It’s impossible for me to do that every meal.

Similar feelings were experienced when the participant tried mySugr Diabetes Diary*.*

#### Different Lifestyle

Different lifestyles lead to different needs. As many older adults eat relatively healthy food, showing *how much junk food have been burnt* (Movesum) was not appealing for them.

While online communities were becoming popular among older adults, joining an online group (Pacer) was not very attractive when they had no idea of using the same app. Few participants checked this function.

The barcode scanner in the *calories calculator and food diary* is designed to reduce the user’s workload of inputting information. However, it can only recognize information on limited packages such as fast food. This design is not in accordance with the lifestyles of older adults who often cook by themselves. One participant said:

I seldom eat fast food. I always cook at home. To get an accurate number of calories, I need to weigh how much the raw material I used in the meal by myself. The scanner won’t help much.

## Discussion

### Principal Findings

This paper has uncovered older adults’ perceptions and initial adoption of mHealth services using qualitative data collected from questionnaires, interviews, and workshops in East London.

Study 1 found that the lack of obvious advantage, low reliability, scary information, and risk of privacy leakage will decrease the *perceived usefulness* of mHealth services; the design of app interface will directly affect the *perceived ease of use*; aging factors, especially the *generation gap*, will make mHealth difficult for older adults to use.

Study 2 identified the barriers that older adults face during their initial adoption of mHealth apps (Figure 3). Access to technology, the way of interaction, the risk of money loss, heavy workload to use an mHealth app, and the different lifestyles of older adults have a great influence on older adults’ adoption of mHealth services.

On the basis of the results of the 2 studies, the implications for the design are summarized in Textbox 1. These suggestions can help design practitioners develop more acceptable mHealth services for a wider population.

**Implications for designing mobile health apps.**

**Technical improvement**

- Reducing the size of the app
- Improving data security
- Improving the accuracy of information
- Providing quick and easy ways of inputting data, especially when similar data are required frequently

**Free trial**

- Providing a free and quick trial of the main service instead of asking for personal information before people know the function of the app

**Information clarification**

- Clarifying what is free and what is paid service
- Making security visible. For example, showing who have access to the data, and explaining why the app needs access to users’ photos or locations
- Providing instruction of the next step, especially when processing takes long
- Avoiding information that scares people

**Participatory design**

- Involving older people in the design process when designing apps that are expected to be adopted by them. Treating them as active consumers of technology [11,49]
- Involving health care professionals in the health information design. The information should be easy for older people to understand

### Implications for Design

The implications fall into 4 categories, that is, technical improvement, free trial, information clarification, and participatory design. The specific suggestions are presented in Textbox 1.

Taking traditional or other existing health care services into consideration and offering (added) advantages.

### Research Contributions

Compared with prior studies, the value of this study lies in its 3 contributions. As for *theoretical contribution*, this study systematically analyzed several main determinants from theoretical models, such as the theory of reasoned action, technology acceptance model, Theory of Planned Behavior, UTAUT2, Health Belief Model, and Protection Motivation Theory, and investigates them through primary research. Some factors were redefined or decomposed according to our results.

*Perceived usefulness* has been used to predict mHealth adoption [16-18,20]. In this paper, *perceived relative advantage* is found to be a better substitute for explaining older adults’ initial adoption of mHealth services. This is in line with the *related advantage* in the diffusion of innovation theory [50]. mHealth services should not only be good but also have more *relative advantage* than traditional health care services. Older adults’ *perceived usefulness* of mHealth services is associated with lifestyle compatibility and information quality. An mHealth service is perceived to be *useful* only when it is compatible with the lifestyle of older adults. This is in accordance with the *compatibility* factor in the diffusion of innovation theory, which indicates how consistent the innovation is with the values, experiences, and needs of potential adopters [50]. Information should not only be *easy to understand* but should also avoid frightening older people.

*Perceived behavioral control* in this paper is investigated through the access to technology and age-related ability decline. *Access to technology* affects older adults’ initial adoption of mHealth apps. Many older adults do not often upgrade their mobile devices or internet services, and their *out-of-date* facilities constrain them from downloading new apps (Figure 3). Age-related ability decline influences older people’s adoption of mobile apps. This is consistent with the findings from previous studies [42,43]. Our study also suggests that *generation gap* creates understanding barriers (Table 8), which has not been addressed by published studies.

*Ease of use* is thought to be perceived firstly from the interface of an App, such as the text and icons (study 1). However, heavy workloads for registration and inputting information often put older people off before they start (study 2).

*Perceived* reliability is positively correlated with the intention to use mHealth services [19]. However, it seems that *the accuracy of the information* is less important in the workshop than what people said in the questionnaire and interview sessions. A novel and easy interaction (eg, using a phone camera to measure blood pressure) can motivate people to start.

mHealth apps generate new security and privacy concerns [51]. Evidence has shown that *perceived risk*, including performance risk, legal concern, and privacy risk, may significantly decrease older people’s intention to use mHealth apps [52]. In our study, the risk of using mHealth apps perceived by the participants was mostly due to privacy leakage (study 1) and unexpected money loss (study 2).

In the *methodological contribution,* the hands-on trial (study 2) illustrates concerns and frustrations when older people *bodily experienced* mHealth apps and provides deeper insights into key issues of initial adoption. The entire study is digitized (access to internet, smartphones, tablets, downloading apps, and initial trial), and goes beyond common technology use among older adults in general.

In the *practical contribution*, we not only investigated perceptions and barriers but also proposed suggestions to design potential barriers. The design implications and specific suggestions are based on the findings of our studies (shown in Textbox 1) to support the better design of mHealth services. Our suggestions share some commonality with [53] which proposed to face cultural resistance and concerns, improve engagement of users in design (see the *participatory design* suggestion in Textbox 1), and build or increase users’ trust (see *free trial* and *information clarification* in Textbox 1). Our more detailed suggestions will help designers tackle these barriers more effectively.

### Limitations and Future Work

This study has several limitations. This research was conducted in East London, and the sampling was not representative of the United Kingdom older population or older adults in general. Participants from different countries and regions could have various perceptions and face different barriers to mHealth services. Gender balance could also have an impact on the results. We tried to balance the participants’ gender, but in reality, study 1 had more male participants (17/30, 57%) and study 2 had more female participants (7/12, 58%). Our participants were relatively well educated, and around 60% were younger *well-old users* (aged 50-60 years). This is because of our recruitment methods and criteria. However, they may be *early adopters* of mHealth services in the future. The workshop participants had limited experience of using mHealth services, which is common among older populations (and given the sampling, the situation of a general older population may be worse). We focused on the initial adoption of mHealth, regardless of the users’ prior experience. It is useful to observe 5 users’ withdrawing from the trial because of the various barriers encountered during the process. Seven users still provide good insights into major usability problems [54,55], and we have been able to learn from both successful and *failed* user-testing.

In our study, eHealth literacy, hedonic motivation, price value, and social influence have not been fully investigated. Future research should address these issues in detail. For future work, more participants with experience using mHealth apps will be recruited to find the motivations in addition to the barriers. Our research was conducted before the COVID-19 pandemic, and health service systems have been largely challenged by the pandemic; significantly more people have experienced remote or web-based health consultation since 2020, which might motivate older adults to accept mHealth if barriers are addressed and trustworthiness is ensured.

## Abbreviations

GP: general practitioner
HBM: Health Belief Model
HM: hedonic motivation
ICT: information and communication technology
PBC: perceived behavioral control
PEOU: perceived ease of use
PHC: perceived healthy condition
PMT: Protection Motivation Theory
PU: perceived usefulness
PV: price value
SI: social influence
TAM: technology acceptance model
TPB: Theory of Planned Behavior
TRA: theory of reasoned action
UTAUT: United Theory of Acceptance and Usage of Technology
